# The Indiana Institute of Homœopathy

**Published:** 1890-07

**Authors:** 


### THE INDIANA INSTITUTE OF HOMŒOPATHY.

The Indiana Institute began its twenty-fourth annual meeting at Indianapolis May 14th, 1890. After prayer by the Rev. E. P. Whallon, a warm welcome was given to the doctors by Dr. O. S. Runnels, who was followed by the President, Dr. J. F. Thompson, with his address.

He made a retrospect of Homœopathy, noting its effect upon medical practice, and also gave a glance as to its future possibilities. He claimed that to Homœopathy is due all the radical modifications of the allopathic school during the last fifty years, while the true homœopathic physician of to-day is following the same fundamental law of cure discovered by Samuel Hahnemann one century ago. That principle is as plain and emphatic now as it ever was. Each one of nature's products, he said, is capable of exerting some influence upon some other of her creations, either good or bad, or, under certain and different circumstances, both good and bad. But nature provides the way in which that influence must be exerted to produce those effects. The same power which gives life takes it again, and the same power which takes life in one instance gives it in another, but both are invariably done according to some law established and laid down by that power. Consequently, if nature fixes by law the amount of a drug or agent necessary to produce death, why, also, does she not fix the amount necessary of such drug or agent to restore a diseased condition, and thereby prolong life?

The bureau of surgery was opened with a paper by Dr. R. St. J. Perry, of Indianapolis, which was discussed up to the noon hour, when adjournment was taken until two P. M., when a report on the condition of Homœopathy in Indiana was made by counties. There are two hundred and thirty homœopathic physicians in the State, and thirty-six county seats without any physicians of this school.

Dr. A. L. Monroe, of Louisville, Ky., President of the delegates from the Kentucky Homœopathic Society, reported that the cause was growing rapidly throughout the entire South. Following this came the reading of a number of papers, among which were "Orificial Work," by Dr. E. W. Viets, Plymouth; "Surgery and Therapeutics," by Dr. J. D. George, Indianapolis; "Anti-Vaccination," by Dr. W. H. Baker, of Terre Haute.

In the evening Dr. G. W. Bowen, of Fort Wayne, opened the clinical bureau with a paper on "Anticipative Treatment," showing how many diseases may be prevented or headed off by certain medicines. This paper was a very interesting one, and was followed by one by Dr. J. R. Haynes, of Indianapolis, on the province and use of certain medicines.

The second day's session began with a paper by Dr. W. H. Baker, of Terre Haute, on "Vaccination," in which he took a decided stand against it. Dr. W. B. Clarke, of Indianapolis, spoke at length, detailing some of the dangers of vaccination as a disease-causer, and as to the sources of impurities. Dr. F. L. Davis, of Evansville, recounted his la grippe cases and their management. Dr. J. S. Mitchell, a distinguished Chicago physician, President of the Homeopathic College in that city, was then introduced as a delegate from the Illinois Society. He responded with a paper detailing his treatment for cancer, a disease of which he claims to have cured many cases. Dr. D. H. Dean, of Columbus, and Dr. D. Clappes, of Mooreland, followed with papers detailing cases of typhoid fever and other diseases. Dr. Alice C. Nivison, of Lafayette, then contributed a valuable paper on "Melancholia." Dr. H. Louis, President of the Missouri Society, was introduced, and responded felicitously as to the condition of Homeopathy in that State, as did Prof. Thomas M. Stewart, of Pulte College, Cincinnati, for the Ohio doctors. Dr. F. L. Davis, of Evansville, contributed a paper on "Materia Medica."

The election of officers for the ensuing year then resulted: President, E. W. Sawyer, Kokomo; First Vice-President, M. H. Waters, Terre Haute; Second Vice-President, W. T. Gott, Crawfordsville; Treasurer, J. S. Martin, Muncie; Secretary, William B. Clarke, Indianapolis.

In the afternoon Dr. I. N. Taylor, of Crawfordsville, President of the State Board of Health, read a paper on "Germ Culture as Related to the Examination of Water," and Dr. M. H. Waters, of Terre Haute, gave a somewhat similar paper on "Germs and their Relation to Disease."

The two papers were interesting and well illustrated. Dr. Taylor was tendered a vote of thanks for his extended labors on the State Board of Health, as reflecting great credit on his Society and School. Dr. W. B. Clarke read a paper on "The Brain Dangers of Quinine," taking the ground that many cases of insanity, suicide, and even murder, are caused by its reckless use. He followed with a short and interesting paper on "Cremation."

Dr. S. J. Hayes, of Pittsburg, then gave a practical illustration of a new and safe method of producing anæsthesia with his apparatus. It was tested on a patient, a little daughter of one of the city physicians, while Dr. W. A. Dunn, of the Hahnemann College, Chicago, a skilled throat operator, performed a difficult operation on the back part of the nose for a serious obstruction. Both were very successful. Dr. Taylor then called attention to an apparent injustice done Prof. S. A. Jones, of the Michigan University, Ann Arbor, several years ago, in dropping him from the rolls of the Institute through an inadvertence. The Society decided that his distinguished services to it and its interests were universally recognized, and it could not afford to be placed in the attitude of even accidentally putting a slight on Dr. Jones, and everything bearing thereon was ordered expunged.

Dr. J. S. Martin, of Muncie, read his paper on "Abrasions of the Cervix," and Dr. L. W. Jordan, of Indianapolis, read one on "Hyperopia," a severe eye trouble, which called out quite a discussion and many inquiries. Other papers were as follows: "Can Criminals be Reformed, or Crimes be Prevented by Medical Treatment?" by Dr. G. W. Bowen, Fort Wayne; "The Philosophy of Homeopathy as Taught by Nature," by Dr. E. P. Jones, Marion; "Professional Hobby-Riders," by Dr. J. E. Mann, Decatur, and "Infantile Convulsions," by Dr. Anna B. Campbell, Rockville. "Divulsion for Stenosis of Cervical Canal," by Dr. O. S. Runnels, Indianapolis; "Obstetrics in Relation to Gynecology," by Dr. E. B. Grosvenor; "Abortion and its Management," by Dr. J. E. Welliver, Rushville; "Puerperal Pelvi-Peritonitis," by Dr. W. D. Hill, Greencastle; "A Bad Confinement That Did Well," by Dr. J. N. Lucas, Shelbyville; "Laryngismus Sicca," by Dr. W. A. Dunn, Wabash; "Atrophic Rhinitis," by Dr. J. N. Taylor, Crawfordsville; "Intubation in Laryngeal Stenosis," by Dr. E. Z. Cole, Michigan City; "Laryngismus Stridulus," by Dr. C. J. F. Ellis, Ligonier.

